# Supplementary material for: The Application of Magnetic Nanoparticles for Sentinel Lymph Node Detection in Clinically Node-Negative Breast Cancer Patients: A Systemic Review and Meta-Analysis
Source: Cancers (Basel). 2022 Oct 14;14(20):5034. doi: 10.3390/cancers14205034 (PMC9599783; doi:10.3390/cancers14205034)
Supplement: Supplementary file 1 [file cancers-14-05034-s001.zip › File S1. Search Strategy in Databases.pdf]

Pubmed

1. Breast Neoplasms[Mesh] OR Breast Neoplasm OR Neoplasm, Breast OR Breast Tumors OR Breast Tumor OR Tumor, Breast OR Tumors, Breast OR Neoplasms, Breast OR Breast Cancer OR Cancer, Breast OR Mammary Cancer OR Cancer, Mammary OR Cancers, Mammary OR Mammary Cancers OR Malignant Neoplasm of Breast OR Breast Malignant Neoplasm OR Breast Malignant Neoplasms OR Malignant Tumor of Breast OR Breast Malignant Tumor OR Breast Malignant Tumors OR Cancer of Breast OR Cancer of the Breast OR Mammary Carcinoma, Human OR Carcinoma, Human Mammary OR Carcinomas, Human Mammary OR Human Mammary Carcinomas OR Mammary Carcinomas, Human OR Human Mammary Carcinoma OR Mammary Neoplasms, Human OR Human Mammary Neoplasm OR Human Mammary Neoplasms OR Neoplasm, Human Mammary OR Neoplasms, Human Mammary OR Mammary Neoplasm, Human OR Breast Carcinoma OR Breast Carcinomas OR Carcinoma, Breast OR Carcinomas, Breast
2. Sentinel Lymph Node [Mesh] OR Lymph Node, Sentinel OR Lymph Nodes, Sentinel OR Sentinel Lymph Nodes OR Sentinal Node OR Node, Sentinal OR Nodes, Sentinal OR Sentinal Nodes
3. Magnetic Iron Oxide Nanoparticles[Mesh] OR Magnetic Iron Nanoparticles OR Iron Nanoparticle, Magnetic OR Magnetic Iron Nanoparticle OR Nanoparticle, Magnetic Iron OR Magnetic IONPs OR IONP, Magnetic OR IONPs, Magnetic OR Magnetic IONP OR Iron Oxide Nanoparticles OR Iron Oxide Nanoparticle OR Nanoparticle, Iron Oxide OR Nanoparticles, Iron Oxide OR Oxide Nanoparticle, Iron OR IONPs OR Hematite Nanoparticles OR Hematite Nanoparticle OR Nanoparticle, Hematite OR Superparamagnetic Iron Oxide Nanoparticles OR Superparamagnetic Iron Nanoparticles OR Iron Nanoparticle, Superparamagnetic OR Nanoparticle, OR Superparamagnetic Iron OR Superparamagnetic Iron Nanoparticle OR SPIONs OR Maghemite Nanoparticles OR Maghemite Nanoparticle OR Nanoparticle, Maghemite
4. 1 AND 2 AND 3

Web of science

#1：TS= (“Breast Neoplasms”* OR “Breast Neoplasm” OR “Neoplasm, Breast”OR “Breast Tumors”

OR “Breast Tumor” OR “Tumor, Breast” OR “Tumors, Breast” OR “Neoplasms, Breast” OR “Breast Cancer” OR “Cancer, Breast” OR “Mammary Cancer” OR “Cancer, Mammary” OR “Cancers, Mammary” OR “Mammary Cancers” OR “Malignant Neoplasm of Breast” OR “Breast Malignant Neoplasm” OR “Breast Malignant Neoplasms” OR “Malignant Tumor of Breast” OR “Breast Malignant Tumor” OR “Breast Malignant Tumors” OR “Cancer of Breast” OR “Cancer of the Breast” OR “Mammary Carcinoma, Human” OR “Carcinoma, Human Mammary” OR “Carcinomas, Human Mammary” OR “Human Mammary Carcinomas” OR “Mammary Carcinomas, Human” OR Human Mammary Carcinoma” OR Mammary Neoplasms, Human” OR “Human Mammary Neoplasm” OR “Human Mammary Neoplasms” OR “Neoplasm, Human Mammary” OR “Neoplasms, Human Mammary” OR “Mammary Neoplasm, Human” OR “Breast Carcinoma” OR “Breast Carcinomas” OR “Carcinoma, Breast” OR “Carcinomas, Breast”)

#2：TS= (“Sentinel Lymph Node” OR “Lymph Node, Sentinel” OR “Lymph Nodes, Sentinel” OR

“Sentinel Lymph Nodes” OR “Sentinal Node” OR “Node, Sentinal” OR “Nodes, Sentinal” OR “Sentinal Nodes”)

#3: TS=(“Magnetic Iron Oxide Nanoparticles” OR “Magnetic Iron Nanoparticles” OR “Iron

Nanoparticle, Magnetic” OR “Magnetic Iron Nanoparticle” OR “Nanoparticle, Magnetic Iron” OR “Magnetic IONPs” OR “IONP, Magnetic” OR “IONPs, Magnetic” OR “Magnetic IONP” OR “Iron Oxide Nanoparticles” OR “Iron Oxide Nanoparticle” OR “Nanoparticle, Iron Oxide” OR “Nanoparticles, Iron Oxide” OR “Oxide Nanoparticle, Iron” OR IONPs OR “Hematite Nanoparticles” OR “ Hematite Nanoparticle” OR “Nanoparticle, Hematite” OR “Superparamagnetic Iron Oxide Nanoparticles” OR “Superparamagnetic Iron Nanoparticles” OR “Iron Nanoparticle, Superparamagnetic” OR “Nanoparticle, Superparamagnetic Iron” OR “Superparamagnetic Iron Nanoparticle” OR SPIONs OR “Maghemite Nanoparticles” OR “Maghemite Nanoparticle” OR “Nanoparticle, Maghemite”)
#4: 1 AND 2 AND 3

Embase

#1. breast tumor/ or breast cancer/
#2. superparamagnetic iron oxide/ or superparamagnetic iron oxide nanoparticle/

#3. sentinel lymph node/ or sentinel lymph node biopsy/
#4. 1 AND 2 AND 3

Cochrane library
#1 Breast Neoplasms*:ME
#2 Sentinel Lymph Node*:ME
#3 Magnetic Iron Oxide Nanoparticles*:ME
#4. 1 AND 2 AND 3
