# Supplementary material for: The Application of Magnetic Nanoparticles for Sentinel Lymph Node Detection in Clinically Node-Negative Breast Cancer Patients: A Systemic Review and Meta-Analysis
Source: Cancers (Basel). 2022 Oct 14;14(20):5034. doi: 10.3390/cancers14205034 (PMC9599783; doi:10.3390/cancers14205034)
Supplement: Supplementary file 1 [file cancers-14-05034-s001.zip › Table S1. Detailed information of the SPIO.pdf]

Table S1. Detailed information of the SPIO in the incorporated studies.

Author, Year	Company	Product name	Concentration	Specification	Doses usage	Detection devices
Rubio et al, 2015[23]	Endomagnetics Ltd, UK	Sienna+	27mg iron per ml	2ml	2ml	(SentimagÒ, Endomagnetics Ltd, UK)
Ahmed et al, 2015[24]	Endomagnetics Ltd, UK	Sienna+	27mg iron per ml	2ml	0.5ml	(SentimagÒ, Endomagnetics Ltd, UK)
Pinero−Madrona et al, 2015[12]	Endomagnetics Ltd, UK	Sienna+	27mg iron per ml	2ml	2ml	(SentimagÒ, Endomagnetics Ltd, UK)
Karakatsanis et al, 2019[25]	Endomagnetics Ltd, UK	Sienna+	27mg iron per ml	2ml	2ml	(SentimagÒ, Endomagnetics Ltd, UK)
Karakatsanis et al, 2018[26]	Endomagnetics Ltd, UK	Sienna+	27mg iron per ml	2ml	2ml	(SentimagÒ, Endomagnetics Ltd, UK)
Karakatsanis et al, 2016[27]	Endomagnetics Ltd, UK	Sienna+	27mg iron per ml	2ml	2ml	(SentimagÒ, Endomagnetics Ltd, UK)
Houpeau et al, 2016[28]	Endomagnetics Ltd, UK	Sienna+	27mg iron per ml	2ml	2ml	(SentimagÒ, Endomagnetics Ltd, UK)
Ghilli et al, 2017[29]	Endomagnetics Ltd, UK	Sienna+	27mg iron per ml	2ml	2ml	(SentimagÒ, Endomagnetics Ltd, UK)
Douek et al, 2013[7]	Endomagnetics Ltd, UK	Sienna+	27mg iron per ml	2ml	2ml	(SentimagÒ, Endomagnetics Ltd, UK)
Alvarado et al, 2019[30]	Endomagnetics Ltd, UK	Sienna+	27mg iron per ml	2ml	2ml	(SentimagÒ, Endomagnetics Ltd, UK)
Thill et al, 2014[14]	Endomagnetics Ltd, UK	Sienna+	27mg iron per ml	2ml	2ml	(SentimagÒ, Endomagnetics Ltd, UK)
Taruno et al, 2019[31]	Resovist; Kyowa CritiCare Co, Ltd, Kanagawa, Japan	Ferucarbotran	27.9mg iron per ml	1ml	1ml	School of Engineering at the University of Tokyo.
Makita et al, 2020[32]	Resovist; Kyowa CritiCare Co, Ltd, Kanagawa, Japan	Ferucarbotran	27.9 mg iron per ml	1ml	0.5ml	School of Engineering at the University of Tokyo.
Rubio et al, 2020[21]	Endomagnetics Ltd, UK	Sienna+	27mg iron per ml	2ml	1.0ml, 1.5ml, or 2ml	(SentimagÒ, Endomagnetics Ltd, UK)
Hamzah et al, 2020[33]	Endomagnetics Ltd, UK	Sienna XP	28mg iron per ml	2ml	2ml	(SentimagÒ, Endomagnetics Ltd, UK)
Hersi et al, 2021[34]	Endomagnetics Ltd, UK	Sienna+	27mg iron per ml	2ml	1.5ml	(SentimagÒ, Endomagnetics Ltd, UK)
Hersi et al, 2021[34]	Endomagnetics Ltd, UK	Sienna+	27mg iron per ml	2ml	1ml	(SentimagÒ, Endomagnetics Ltd, UK)
Giménez-Climent et al, 2021[35]	Endomagnetics Ltd, UK	Sienna+	27mg iron per ml	2ml	2ml	(SentimagÒ, Endomagnetics Ltd, UK)
Vidya et al, 2022[36]	Endomagnetics Ltd, UK	Sienna+	27mg iron per ml	2ml	2ml	(SentimagÒ, Endomagnetics Ltd, UK)
