# Supplementary material for: The Application of Magnetic Nanoparticles for Sentinel Lymph Node Detection in Clinically Node-Negative Breast Cancer Patients: A Systemic Review and Meta-Analysis
Source: Cancers (Basel). 2022 Oct 14;14(20):5034. doi: 10.3390/cancers14205034 (PMC9599783; doi:10.3390/cancers14205034)
Supplement: Supplementary file 1 [file cancers-14-05034-s001.zip › Table S2. ROBINS-I.pdf]

Table S2. Risk of bias assessment.

Author and Year	Bias due to confounding	Bias in selection of participants into the study	Bias in classification of interventions	Bias due to deviations from intended interventions	Bias due to missing data	Bias in measurement of outcomes	Bias in selection of the reported result	Overall
Rubio et al, 2015[21]	Low	Low	Low	Low	Low	Low	Middle	Middle
Ahmed et al, 2015[15]	Low	Low	Low	Low	Low	Low	Low	Low
Pinero−Madrona et al, 2015[20]	Low	Low	Low	Low	Low	Low	Low	Low
Karakatsanis et al, 2019[27]	Low	Low	Low	Low	Low	Low	Low	Middle
Karakatsanis et al, 2018[25]	Middle	Low	Low	Low	Low	Low	Low	Middle
Karakatsanis et al, 2016[23]	Low	Low	Low	Low	Low	Middle	Low	Middle
Houpeau et al, 2016[22]	Low	Low	Low	Low	Low	Low	Low	Low
Ghilli et al, 2017[24]	Low	Low	Low	Low	Low	Low	Low	Low
Douek et al, 2013[29]	Low	Low	Low	Low	Low	Low	Low	Low
Alvarado et al, 2019[26]	Low	Low	Low	Low	Low	Low	Low	Low
Thill et al, 2014[19]	Low	Low	Low	Low	Low	Low	Low	Low
Taruno et al, 2019[17]	Low	Low	Low	Low	Low	Middle	Low	Middle
Makita et al, 2020[16]	Low	Low	Low	Low	Low	Low	Low	Low
Rubio et al, 2020[13]	Low	Low	Low	Low	Low	Low	Low	Low
Hamzah et al, 2020[28]	Low	Low	Low	Low	Low	Middle	Low	Middle
Hersi et al, 2021[18]	Low	Low	Low	Low	Low	Low	Low	Low
Hersi et al, 2021[18]	Low	Low	Low	Low	Low	Low	Low	Low
Giménez-Climent et al, 2021[30]	Low	Low	Low	Low	Low	Low	Low	Low
Vidya et al, 2022[31]	Low	Low	Low	Low	Low	Low	Low	Low
