# Supplementary figures and images for: The Application of Magnetic Nanoparticles for Sentinel Lymph Node Detection in Clinically Node-Negative Breast Cancer Patients: A Systemic Review and Meta-Analysis
Source: Cancers (Basel). 2022 Oct 14;14(20):5034. doi: 10.3390/cancers14205034 (PMC9599783; doi:10.3390/cancers14205034)

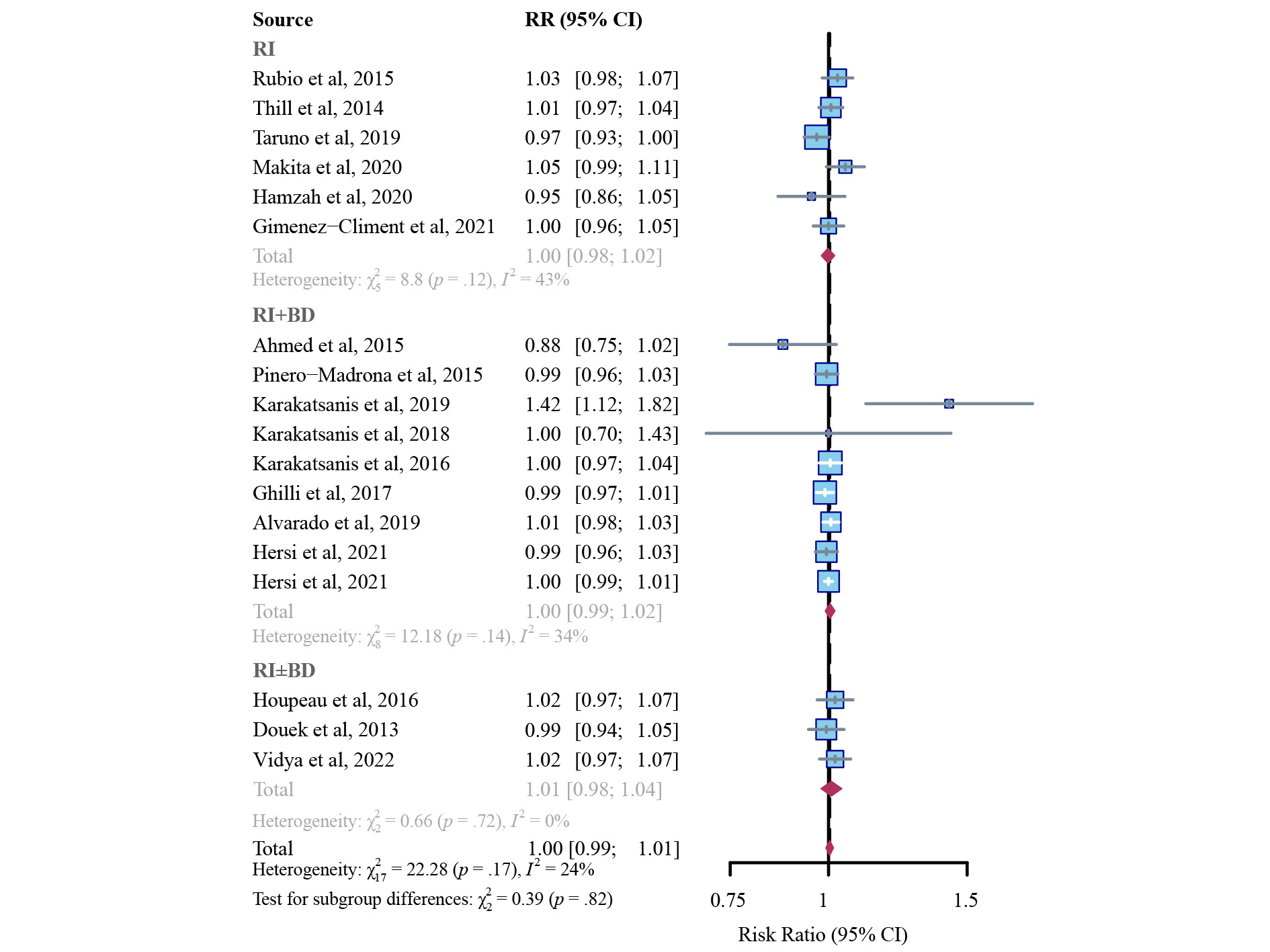

Supplement: Supplementary file 1 [file cancers-14-05034-s001.zip › Figure S1.jpg]

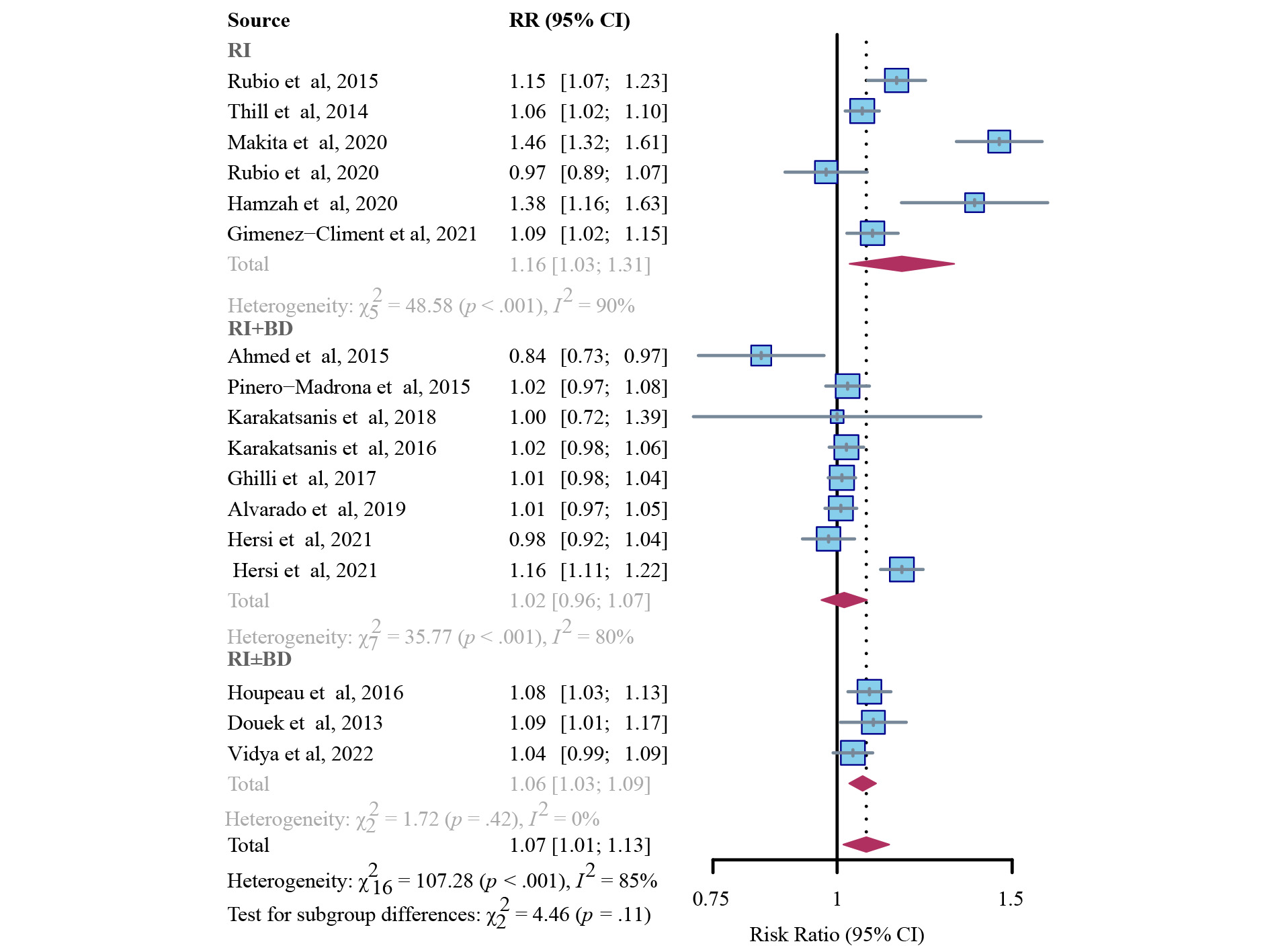

Supplement: Supplementary file 1 [file cancers-14-05034-s001.zip › Figure S2.jpg]
